# Proximal Tibia Hemiarthroplasty Reconstruction Following Resection of Malignant Bone Tumors in Skeletally Immature Patients

**DOI:** 10.1016/j.jposna.2024.100118

**Published:** 2024-09-16

**Authors:** Tyler Kelly, Lee J. Morse, Rosanna Wustrack, Melissa Zimel

**Affiliations:** 1 School of Medicine, University of South Carolina, Greenville, SC, USA; 2 Department of Orthopaedic Surgery, Kaiser Oakland Medical Center, Oakland, CA, USA; 3 Department of Orthopaedic Surgery, University of California San Francisco, CA, USA

**Keywords:** Osteosarcoma, Pediatric limb salvage, Expandable prostheses, Tibia hemiarthroplasty

## Abstract

**Background:**

Reconstruction of the proximal tibia following resection of malignant bone tumors in pediatric patients is traditionally limited to osteoarticular allografts or endoprostheses. Endoprostheses typically require resection or disruption of the distal femoral physis, which can lead to a leg length discrepancy (LLD). We introduce a novel form of proximal tibia limb reconstruction using a Compress® tibia hemiarthroplasty, which spares the distal femoral physis.

**Methods:**

We retrospectively reviewed 5 patients who underwent proximal tibia osteosarcoma resection and reconstruction with a custom Compress® proximal tibia hemiarthroplasty. Data on function, survival, LLD, complications, and patient-reported outcomes were collected.

**Results:**

Mean age at resection was 10.4 years [range: 8.8-12.9 years]. Mean implant survival was 59 months [range: 34-83 months]. One patient developed a deep infection, and two patients required implant lengthening. Both were later converted to a rotating hinged total knee arthroplasty (RHTKA) ​> ​58 months after index surgery. At the last follow-up, all living patients had knee range of motion from 0 to 110°, walked unassisted, and had no LLD or knee instability. Mean Toronto Extremity Salvage Score was 90 [range: 80-97].

**Conclusions:**

Proximal tibia hemiarthroplasty reconstruction after tumor resection in skeletally immature patients preserves the distal femoral physis and may potentially reduce LLD and delay conversion to an RHTKA until after skeletal maturity.

**Key Concepts:**

(1) Osteosarcoma is the most common primary malignant bone tumor in children, arising most frequently around the knee.
(2) Complete resection often requires excising the adjacent growth plate, creating a challenge for reconstruction in growing children to maintain function and avoid significant limb length inequality.
(3) The custom expandable tibia hemiarthroplasty is a novel reconstruction option for skeletally immature patients requiring resection of the proximal tibia.
(4) Although future research is needed, results of this study suggest that tibia hemiarthroplasty is a reasonable reconstruction option in growing children requiring oncologic resection of a primary bone tumor from the proximal tibia.

**Level of Evidence:**

Case series, Level IV

## Introduction

High-grade osteosarcoma is the most common primary malignant bone tumor in childhood, arising most frequently in the distal femur or proximal tibia, and has a 70% 5-year overall survival for patients presenting with a localized disease [[1], [2], [3]]. Following neoadjuvant chemotherapy, wide excision with limb-salvage surgery is performed in nearly 90% of patients, with amputation or rotationplasty only utilized in select cases [4,5]. Despite advances in limb-salvage techniques, osteosarcoma often abuts, invades, or even crosses the physis, requiring resection of epiphysis to obtain a negative margin [4,6]. Thus, in skeletally immature patients, a reconstruction technique that can potentially match future longitudinal bone growth of the contralateral limb should be considered in order to avoid a clinically significant leg length discrepancy (LLD), traditionally classified as greater than 2 ​cm [7,8]. Furthermore, the limb reconstruction method should be durable, restore mobility, and allow for continuation of daily function [1,2,4].

For skeletally immature patients with a malignant tumor in the proximal tibia, reconstruction options have traditionally been limited to osteoarticular allografts [9], static or expandable endoprostheses that is coupled with a rotating platform total knee arthroplasty [10], or allograft prosthetic composites [1,2,11]. Osteoarticular allografts do spare the distal femoral physis but are static and subject to complications such as fracture, LLD, and arthrosis of the adjacent knee joint and frequently end with revision to an endoprosthesis [12,13]. Expandable endoprostheses allow for minimally invasive or noninvasive lengthening to match the length of the contralateral limb but do disrupt the physis on the other side of the joint as they require fixation for the rotating hinge knee mechanism [4,[14], [15], [16], [17]]. Allograft prosthetic composites are rarely described in the pediatric limb-salvage literature and may also be used less frequently, given their static length [11]. Lastly, there is also a reported technique of proximal tibia epiphyseal distraction for pediatric patients with malignant bone tumors, but this can only be considered if the tumor has not crossed the physis [18].

Expandable endoprostheses are commonly used for limb reconstruction around the knee in very young patients as they can expand to match the contralateral growing limb [1,4,[14], [15], [16], [17],[19], [20], [21]]. Since the approximate growth per year from distal femoral physis is 9 ​mm and growth from the proximal tibial physis is 6 ​mm, resection of the proximal tibia physis, coupled with resection or disruption of the distal femur physis, increases the potential for a clinically significant LLD [4,14,15]. Expandable implants require repeated lengthening procedures and have a finite length of expansion. Once this limit is reached, a new implant must be exchanged if further length is required. These lengthening procedures place patients at an increased risk for complications such as infection and arthrofibrosis, or even subsequent amputation [16,17,[19], [20], [21], [22]]. In the United States, the two commercially available proximal tibia replacement endoprostheses with a rotating hinged total knee arthroplasty (RHTKA) require either resection of the distal femoral physis (Orthopedic Salvage System; Zimmer Biomet, Warsaw, IN, USA) or disruption of the distal femoral physis (Global Modular Replacement System; Stryker, Mahwah, NJ, USA). The Juvenile Tumor System (ONKOS, Parsippany, NJ, USA) provides potential for noninvasive expansion and also disrupts the distal femur physis with reported subsequent leg length discrepancies [14,15,17,19]. Therefore, if the distal femoral physis can be left intact and an expandable proximal tibia endoprosthesis can be utilized, LLD and other complications in a growing child may be reduced.

In the current medical literature, very few proximal tibia endoprosthesis limb-salvage reconstruction options are described that spare the distal femur. Tibia hemiarthroplasty constructs are scarce and are often limited to case reports [[25], [26], [27]]. One biomechanical study examined the concept of knee hemiarthroplasty [19]. Cement spacer constructs and the use of a cemented tibia stem in pedicle frozen tumor were also described, all using a cemented stem for fixation [25,26,28]. The primary aim of this study was to investigate a novel form of proximal tibia endoprosthetic reconstruction using a custom Compress® tibia expandable hemiarthroplasty. The Compress® Compliant Pre-stress (CPS) implant (Zimmer Biomet, Warsaw, IN, USA) has excellent long-term survivorship at the bone-implant interface: 85% at 5 years and 80% at 10 years in one published report [28]. The implant is fixed to the bone with compressive osseointegration at the implant-bone interface, with multiple other studies reporting excellent survivorship without aseptic mechanical failures in greater than 93% of patients at 4 years and greater than 88% at 10 years [[29], [30], [31]]. Furthermore, the anchor plug length of 5 ​cm allows for more preservation of bone than a typical stemmed implant, which is ideal when the residual bone length is limited.

Given the demonstrated durability of this implant fixation device, a custom expandable tibia hemiarthroplasty implant was mated to the CPS spindle, allowing for preservation of the distal femoral physis and for future minimally invasive implant expansion if needed. We hypothesized that this reconstruction method is an acceptable option for proximal tibia reconstruction in skeletally immature patients and that preservation of the distal femoral physis would decrease the incidence of significant limb length discrepancy. In addition, we report associated complications with this procedure and functional outcomes that suggest this reconstruction technique should be considered for reconstruction in growing children who require oncologic resection of a primary bone tumor from the proximal tibia.

## Materials and methods

We conducted a retrospective chart review of 5 pediatric patients who underwent proximal tibia tumor resection and reconstruction with a custom Compress® expandable tibia hemiarthroplasty reconstruction between 2015 and 2019. This review was conducted under an institutional review board–approved research study protocol. The procedures were performed by three orthopaedic surgical oncologists practicing at two institutions. All patients received neoadjuvant chemotherapy per Children's Oncology Group protocol [32], local control with wide surgical resection, and adjuvant chemotherapy starting 3 weeks after surgery. Patients had regularly scheduled follow-up visits with a clinical examination and radiographs at 3 weeks, 6 weeks, 12 weeks, and then every 3 months for the first 2 years. Clinical documentation from these follow-up appointments was reviewed to obtain data on patient demographics, implant survival, LLD, knee stability, knee range of motion (ROM), as well as additional surgeries and complications. Implant survival was calculated from the time of index surgery to the time of implant removal or last follow-up. The Toronto Extremity Salvage Score (TESS) [33] was administered to patients at the most recent follow-up to obtain patient-reported functional outcomes. Mean scores were calculated, but no further statistical analysis was carried out, given the small patient sample.

### Surgical indications

Once the diagnosis of a primary malignant bone tumor in a pediatric patient is confirmed, a discussion regarding the nature of the surgical resection and method of reconstruction is held with the patient and their family. Patients with greater than 2 years of future potential limb growth, or more than 3 ​cm of future limb length, are considered candidates for an expandable implant. Patients meet these criteria if they had open physes and/or were prepubescent. This included females aged 8 to 13 years and males aged 8 to 15 years. Older pediatric patients, or those with closed physes, who require endoprosthetic reconstruction receive a static implant. Very young patients, under 8 years, may require amputation, rotationplasty, or alternative biologic reconstruction, given their small size. The decision to resect the epiphysis of the tibia with the tumor is made by the surgeon 2 cm with the goal of achieving a wide bony surgical margin of at least two centimeters.

### Custom implant specifications

Within 2 weeks of diagnosis of a proximal tibia osteosarcoma, local control options were discussed with the patient and their parents. In young patients with greater than 3 ​cm of anticipated future longitudinal growth, whose families elected limb salvage, they were presented with this novel method of reconstruction. Specifications for the custom tibia CPS hemiarthroplasty implant were then provided to the implant manufacturer (Zimmer Biomet, Warsaw, IN, USA). All implants used a non-custom short (5 ​cm) CPS anchor plug, spindle, and taper adaptor. The minimum tibia resection length to accommodate this implant is 15 ​cm, including the 1-cm spindle and 5-cm taper adaptor. The maximum amount of expansion allowed with this design was 3 ​cm. Implant expansion is achieved by engaging the screwdriver into the side of the implant body and turning it manually to lengthen the implant. This requires a small incision and is performed under fluoroscopic guidance with the patient under anesthesia. The implant is typically lengthened 1 ​cm during this procedure to avoid excess stretch on nerves and blood vessels. The expandable tibia hemiarthroplasty component was a custom implant approved for manufacturing under a Food and Drug Administration compassionate use device exemption. The manufacturing time varied from 7 to 8 weeks. The co-authors of this paper and participating surgeons in this case series have no financial or consulting relationships with the implant manufacturer.

### Surgical technique

Wide surgical excision of the proximal tibia osteosarcoma was performed, resecting the tibia epiphysis and a minimum of 2 ​cm of bone distal to the tumor, along with a soft tissue envelope overlying the tumor, sufficient to obtain a grossly negative margin. The medial collateral ligament (MCL), lateral collateral ligament (LCL), anterior cruciate ligament (ACL), posterior cruciate ligament (PCL), and the patellar tendon were transected 1 ​cm from their respective attachments on the proximal tibia and marked with suture for future repair. The joint capsule was transected 1 ​cm proximal to the tibia articular surface.

The custom CPS expandable tibia hemiarthroplasty implant length was predetermined based on initial magnetic resonance imaging obtained at the time of diagnosis. A tibial osteotomy was made at a distance from the joint line to match the length of the custom implant. CPS anchor plug, spindle, and taper adaptor were standard components and were attached to the tibia diaphysis following the manufacturer’s technique guide. The custom implant was then attached to the taper adaptor. A custom highly cross-linked polyethylene was designed to match the contour of the patient’s distal femoral condyles with a posterior stabilizing post. The post was intended to limit anterior translation of the implant with respect to the femur. This implant was also designed to allow for future conversion to a rotating hinged total knee arthroplasty without need for removal of the proximal tibia implant.

**Figure 1:**
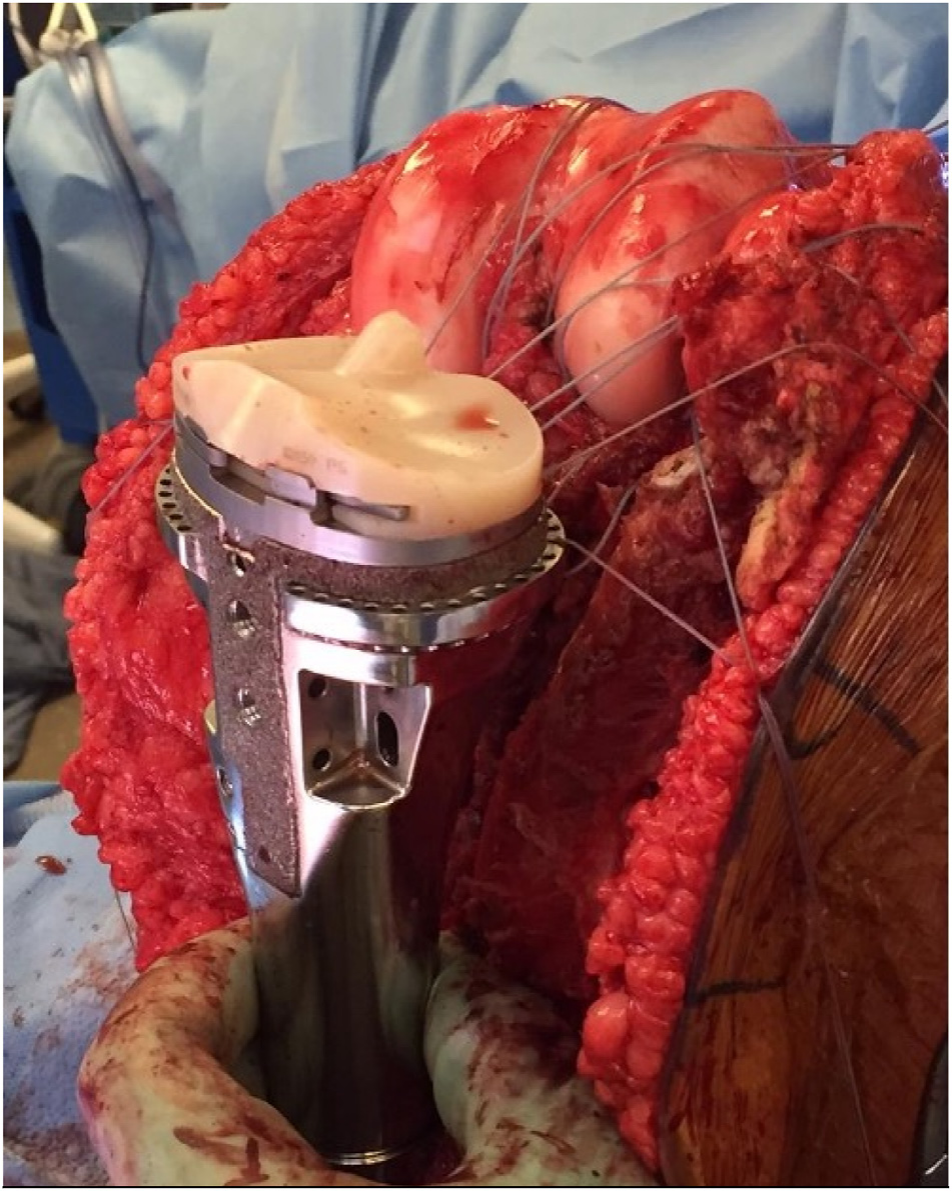
Intraoperative photograph of proximal tibia hemiarthroplasty with suture connecting posterior capsule, PCL, MCL, and ACL to the implant. ACL, anterior cruciate ligament; MCL, medial collateral ligament; PCL, posterior cruciate ligament

**Figure 2:**
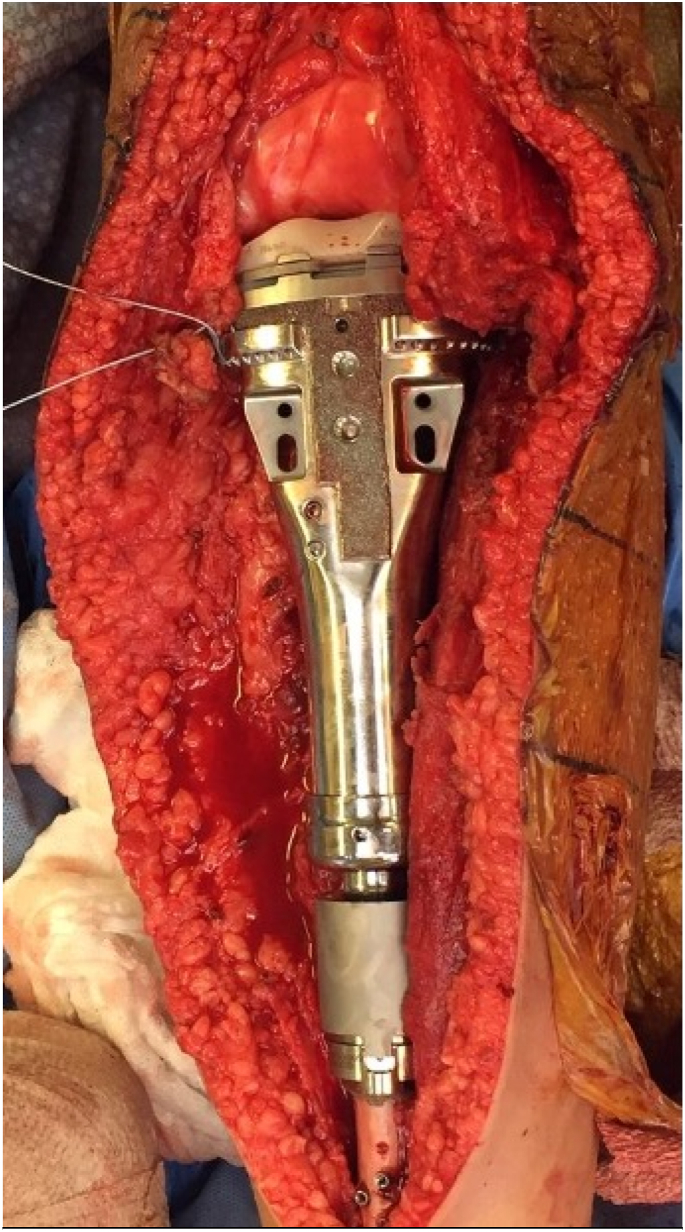
Intraoperative photograph of knee in extension with posterior, medial, and lateral structures tied to the implant.

The proximal aspect of the custom implant has circumferential premade holes allowing for attachment of the collateral ligaments, cruciate ligaments, and capsule. Additional anterior holes are placed for attachment of the patellar tendon. After resection of the tumor and attachment of the implant to the residual tibia, the soft tissue reconstruction is performed. With the knee in flexion, the posterior capsule and PCL are tightly secured to the posterior implant holes with braided, nondissolvable suture (Fig. 1). Next, the knee is brought into 10 degrees of flexion and the lateral and medial capsule, LCL, and MCL are then sewn to the implant with equal tension (Fig. 2). Lastly, the ACL and anterior capsule are attached and tightened with the knee fully extended. The patellar tendon is then attached to anterior holes in the proximal body of the implant. A medial gastrocnemius flap is then harvested, rotated to cover the extensor mechanism, and proximally oversewn [34]. One proximal and one distal drain are placed, and the wound is closed in a layered fashion. A split thickness skin graft is then harvested, if needed, to cover the gastrocnemius flap.

### Postoperative protocol

Patients are restricted to non-weight-bearing or toe-touch weight-bearing on their surgical extremity for 12 weeks, per surgeon’s preference. Patients were placed in a hinged knee brace locked in extension for 8–12 weeks, after which they are permitted to perform graduated knee ROM exercises. At 12 weeks following surgery, they are advanced to 25% weight-bearing and then increase to 50% weight-bearing at 14 weeks. At 16 weeks, they advance to 75% weight-bearing and are allowed to fully weight-bear at 18 weeks. The hinged knee brace is worn, unlocked for the first 3 months of full weight-bearing, after which it is discontinued. Scanograms, or standing full-leg radiographs, were performed annually following the index procedure, until the patient’s ipsilateral distal femur physis and contralateral physes were closed. If the measured limb difference was greater than or equal to 2 ​cm, an implant lengthening was offered to the patient. Implants were lengthened 1 ​cm per procedure. A patient whose scanogram demonstrated a measured overall limb difference of less than 5 ​mm was considered to have equal limb lengths.

## Results

A retrospective chart review identified 5 skeletally immature patients, all diagnosed with a proximal tibia high-grade osteosarcoma, who underwent tumor resection and reconstruction with a custom CPS expandable tibia hemiarthroplasty between 2015 and 2019. Patient demographics and functional outcomes are detailed in Table 1.

**Table 1:** Patient demographics and outcomes.

**Case**	**Sex**	**Age at surgery (years)**	**Resection length (mm)**	**Implant survival (months)**	**Active knee ROM at last f/u**	**Additional surgeries**	**Type**	**LLD**	**TESS**
1	F	8.8	150	65.5	0-130°	3	L, L, C	None	91

2	M	12.3	170	38.1	0-15-100°	3	L, I, C	None	80

3	F	9.3	150	67.0	0-110°			None	97

4	F	8.7	170	34.5∗	0-95°			15 ​mm	88

5	F	12.9	170	52.4	0-110°			None	95

Mean		10.4	162	51.5					90

*f/u, follow-up; ROM, range of motion; LLD, leg length discrepancy; TESS, Toronto Extremity Salvage Score*.

**Figure 3:**
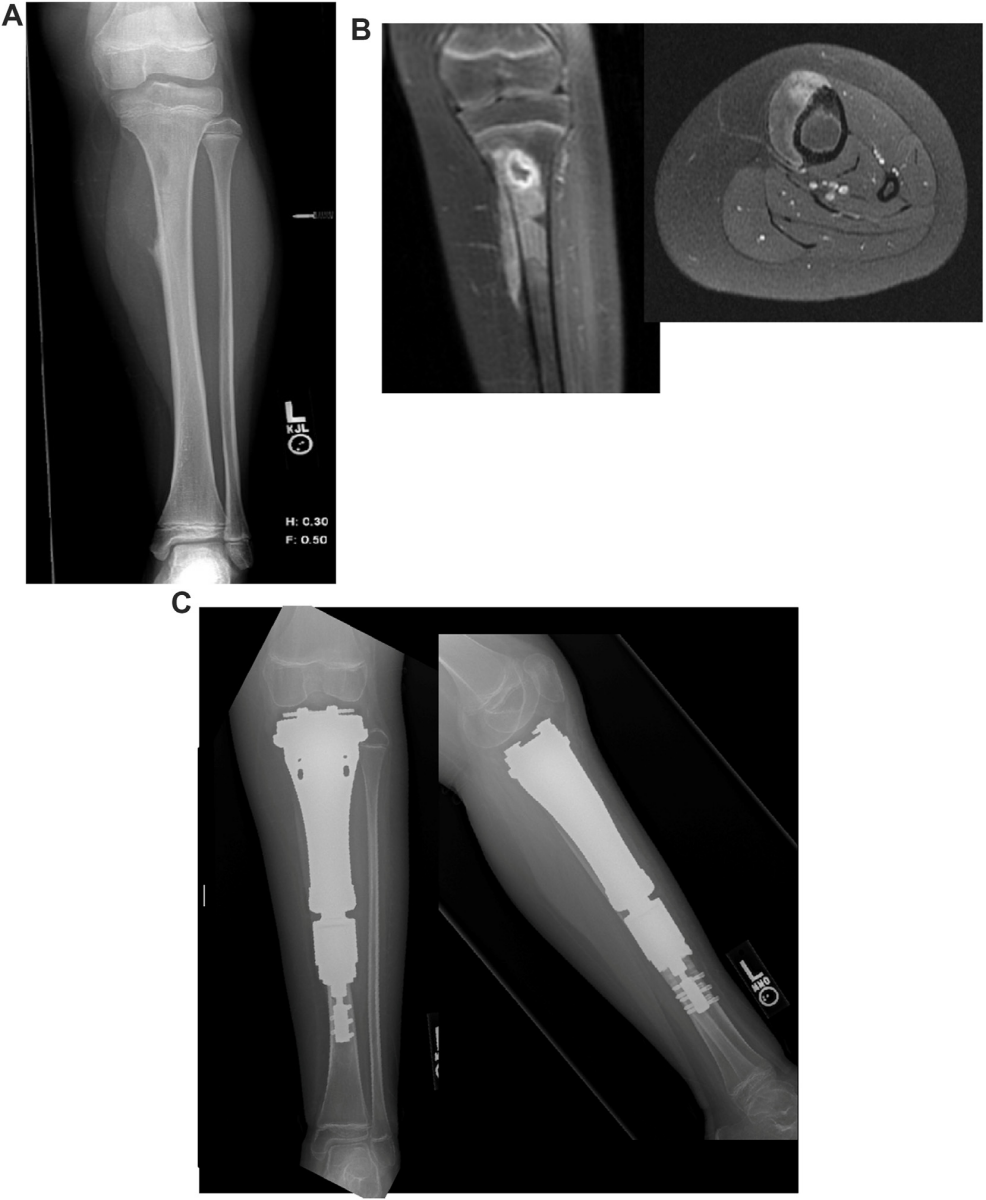
A. AP radiographs of an eight-year-old female with proximal tibia osteosarcoma. B. Post-gadolinium MR coronal and axial images obtained after administration of neoadjuvant chemotherapy showing residual disease. C. Postoperative AP and lateral radiographs following reconstruction with the custom proximal tibia hemiarthroplasty implant. AP, anteroposterior; MR, magnetic resonance.

There were 4 females and 1 male. The mean age at index surgery was 10 years, 5 months [range: 8.8-12.9 years]. The mean tibia resection length was 162 ​mm [range: 150-170 ​mm]. Fig. 3 shows the **(**A**.)** preoperative radiograph, **(**B**.)** MRI, and **(**C**.)** postoperative radiographs of case 1 in the series. Resection of the epiphysis was performed, given the proximity of the tumor to the physis.

**Figure 4:**
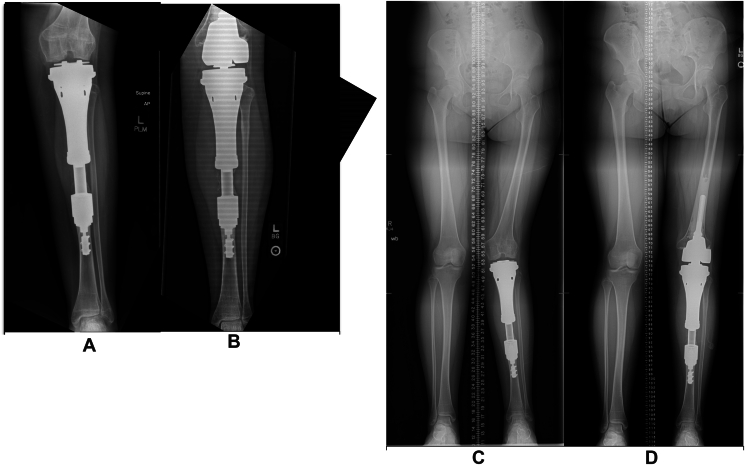
A. Prerevision radiograph showing condyle wear. B. Postrevision radiograph. C. Prerevision scanogram showing knee valgus deformity. D. Postrevision scanogram.

The mean implant survival was 59 months [range: 35-83 months]. Two of the five patients required additional surgical procedures. The first patient (case 1) had 2 implant lengthening procedures, 1 year apart, after the scanogram revealed an LLD of 2 ​cm. She was then converted to a RHTKA 66 months after index surgery for valgus instability, pain, and radiographic signs of erosion of the lateral femoral condyle, at which time her physes were closed. Intraoperatively, her lateral condyle cartilage wear was grossly visible. The hemiarthroplasty polyethylene did not exhibit any signs of wear. Her CPS spindle, anchor plug, and proximal tibia placed at the index procedure were retained. Fig. 4A and B show the AP tibia radiographs before and after revision to an RHTKA. C and D show the scanograms before and after revision to the RHTKA.

The second patient (case 2) requiring multiple procedures underwent a lengthening procedure for a 2-cm LLD on scanogram. The implant was lengthened 1 ​cm. Unfortunately, 1 month later, he developed a periprosthetic infection requiring removal of the implant at 38 months after the index procedure. He was treated with an antibiotic spacer and then converted to an RHTKA 58 months after index surgery, at which time his physes were closed. His CPS spindle and anchor plug were retained, given intact osseointegration or bony fixation at the bone-implant interface. The remaining components, including the hemiarthroplasty component, were replaced, given the prior infection. These two patients converted to an RHTKA are now 30 and 36 months out from the revision surgery. They walk unassisted and have equal leg lengths, or a <5 ​mm measured difference in overall limb length on the final scanogram. Case 2 has no evidence of recurrent periprosthetic infection.

The remaining 3 patients had no additional procedures. One patient died from metastatic disease 35 months after index surgery and had an LLD of 1.5 ​cm at their last follow-up visit measured on scanogram. The final two patients are alive with their original implant and have required no additional procedures at 75 and 83 months from index surgery. They both walk unassisted and do not have any knee instability. On the last scanogram demonstrating closed physes, they had overall leg length measurements within 2 ​mm. For all 5 patients, at the last follow-up, knee ROM was 0 to 90-120°. The mean TESS at the last follow-up visit was 90 [80–97].

## Discussion

Reconstruction of the proximal tibia in skeletally immature patients, following resection of a malignant bone tumor, presents the unique challenges of matching future limb growth of the contralateral limb while creating a durable construct that will allow for maintenance of function. Numerous publications describe reconstruction methods around the knee in pediatric patients with primary bone sarcomas [1,[4], [5], [6],[9], [10], [11], [12], [13], [14], [15], [16], [17], [18]]. Complications can arise when reconstructing the proximal tibia with either an osteoarticular allograft or an expandable endoprosthesis and have been well documented in the literature [[13], [14], [15], [16], [17],[19], [20], [21], [22]]. There is a need for continued innovation and research to improve the outcomes for young children undergoing limb salvage.

This study reports the results of a novel method used to reconstruct proximal tibia oncologic defects that spares the distal femoral physis and allows for expansion of the implant to match the longitudinal growth of the contralateral limb. Since the distal femur physis is not resected or disturbed, there is a hypothetical reduced risk of developing a clinically significant LLD, generally defined as greater than 2 ​cm. Two of the patients in this cohort did have implant lengthening and subsequent revision to an RHTKA and now have equal leg lengths, or less than a 5-mm difference in overall limb lengths on the last scanogram after all physes had closed. One patient died from the disease with a 1.5-cm LLD. The other 2 did not require lengthening and have a less than 2-mm difference in overall limb length on the last scanogram, attributed to ipsilateral femur overgrowth in one patient and limited contralateral tibia growth in the other patient. Epiphysiodesis was not performed or considered in any of the patients. Idowu et al. [15] described the femoral bone growth after reconstruction with a proximal tibia replacement RHTKA where the distal femoral physis is disrupted by the implant stem, but not resected. In 12 patients, distal femoral physis growth continued, but 4 (33%) patients had a >2-cm LLD and 6 (50%) patients had a femoral discrepancy of >2 ​cm. This suggests that the physeal disruption from implant fixation may not be ideal with respect to future potential limb length in a growing child.

After an extensive literature search for other tibia hemiarthroplasty constructs, only a few studies were identified. Chung et al. [24] described the results of 13 temporary tibial hemiarthroplasties performed as part of a 3-staged surgery. A revision long-stemmed tibia component and cement composite was placed at the time of tumor resection. Once the patient developed an LLD of >5 ​cm, their provisional implant was removed, and the soft tissues were lengthened with an Ilizarov frame. Once leg lengths were even, a nonexpandable proximal tibia RHTKA was placed. The study reported 3 failures necessitating knee fusion. Patients without failure underwent >3 surgeries with complications including significant LLDs and infections. Average ROM was 60-130° [24]. Lozano-Calderon and Kenan [26] described 3 pediatric patients who received a total condylar unipolar expandable prosthesis. This implant was fixed to the residual tibia with a press-fit stem. One patient had arthrofibrosis, and one patient died due to a secondary malignancy. No long-term outcomes are reported.

Functional outcomes for the current series of five patients are favorable compared with patients in the aforementioned proximal tibia limb-salvage studies. At a follow-up ranging from nearly 3 to 6.9 years, all surviving patients were ambulatory without an assistive device. One patient died from metastatic disease 35 months after surgery. Two patients have not had any additional procedures at 75 and 83 months from the index procedure. Only 2 patients underwent additional lengthening procedures. Both patients wore a hinged knee brace for valgus laxity and did have radiographic signs of lateral femoral condylar erosion. Unfortunately, one of these patients developed a periprosthetic infection 1 month after a lengthening procedure, necessitating implant removal and placement of a cement-coated spacer. He was converted to an RHTKA and is now doing well 2.5 years later. The second patient required two additional lengthening procedures, after which she was converted to an RHTKA for valgus instability and symptomatic erosion of her lateral condyle, now 3 years ago, and is also doing well. All four living patients, including the two converted to an RHTKA, retained their tibial CPS spindle and had radiographic evidence of osseointegration at the spindle-bone interface. The patients have not suffered any implant failures or soft tissue complications. Average knee ROM 0-110° reflects the lack of arthrofibrosis in this small group. A mean TESS of 90 reflects the ability of these young patients to perform daily activities without difficulty.

The custom CPS expandable tibia hemiarthroplasty offers potential advantages where other reconstruction options are limited. CPS implant fixation has demonstrated excellent long-term survivorship at the bone-implant interface, equal to or exceeding that of cemented stem fixation which is more prone to aseptic loosening [[28], [29], [30], [31]]. The CPS anchor plug is only 5 ​cm in length, which requires less bone to anchor the implant than a traditional stemmed implant. All 5 patients retained their original CPS spindle, even if converted to an RHTKA, and had no evidence of aseptic loosening on the most recent radiographs.

In our limited series of patients with custom expandable tibia hemiarthroplasty implants, 0-2 lengthening procedures were performed per patient. In other publications describing expandable implants, where resection or disruption of the distal femoral physis is required for RHTKA fixation, a higher number of implant expansions were reported per patient. Picardo et al. [14] described a series of 55 patients who underwent a mean 11.3 [range: 1-40] noninvasive lengthenings of either distal femur or proximal tibia expandable endoprostheses. One could hypothesize that fewer minimally invasive lengthenings may decrease the number of opportunities for a periprosthetic infection, a known complication of expandable megaprostheses [14,17,20,21]. However, Tsagozis et al. [20] did report a higher rate of infections in prostheses with noninvasive lengthening than in conventional implants that require a minimally invasive lengthening. They attributed these data to the noninvasive expandable implant being bulkier in an area with generally poor soft tissue coverage. Grimer et al. [17] reported an overall risk of infection of 68% within 10 years, and the risk per lengthening was 5.1% for proximal tibia expandable implants. Our cohort is too small to generalize our infection rate of 20% or one patient or find an association between the number of minimally invasive implant lengthenings and subsequent outcomes. Furthermore, a contralateral proximal tibia epiphysiodesis could have been considered instead of lengthening the proximal tibia implant, but this was not performed. Only 2 of the 5 patients had their implant lengthened prior to conversion to an RHTKA. The other two are skeletally mature and did not undergo lengthening. Thus, we cannot determine the ultimate benefit of placing an expandable tibia hemiarthroplasty implant versus preservation of the distal femur physis alone.

This case series does have significant limitations. First, there are only 5 patients, one with only 35-month follow-up, given death from disease. Longitudinal follow-up and inclusion of more patients is critical to better assess the durability of this construct and long-term outcomes. Only one biomechanical study looked at possible wear mechanisms of a tibia hemiarthroplasty construct [23]. More data are needed to understand the long-term wear pattern and subsequent clinical consequences of polyethylene wear on the cartilage of the distal femoral condyle. Ultimately, further innovation and collaborative research is required to improve both systemic therapy for malignant bone tumors and reconstruction methods that improve quality of life and maintain function after limb salvage for pediatric patients.

## Conclusion

The current results of this study suggest that a CPS expandable proximal tibia hemiarthroplasty may be an acceptable alternative to static osteoarticular allografts or expandable proximal tibia replacement RHTKA endoprostheses that require disruption or resection of the distal femoral physis for patients with more than 3 ​cm of future potential limb growth. Our small series of patients demonstrate that proximal tibia hemiarthroplasty reconstruction, after tumor resection in a skeletally immature patient, is a possible alternative surgical technique to preserve the distal femoral physis, potentially lessen LLD, and delay conversion to an RHTKA until after skeletal maturity.

## Consent for publication

Complete written informed consent was obtained from the patient, their guardian, or legal representative for the publication of this study and accompanying images.

## Author contributions

**Tyler Kelly:** Writing – review & editing, Writing – original draft, Formal analysis, Data curation. **Lee J. Morse:** Writing – review & editing, Data curation, Conceptualization. **Rosanna Wustrack:** Writing – review & editing, Formal analysis, Conceptualization. **Melissa Zimel:** Writing – review & editing, Writing – original draft, Supervision, Project administration, Methodology, Investigation, Formal analysis, Data curation.

## Funding

There was no funding received for this original research.

## Declaration of competing interests

The authors declare that they have no known competing financial interests or personal relationships that could have appeared to influence the work reported in this paper.
